# In Reply

**DOI:** 10.1093/oncolo/oyac058

**Published:** 2022-03-24

**Authors:** Karan Rai, Gabriel Brooks

**Affiliations:** 1 Geisel School of Medicine at Dartmouth, Lebanon, NH, USA; 2 The Dartmouth Institute for Health Policy and Clinical Practice, Geisel School of Medicine, Lebanon, NH, USA; 3 Dartmouth-Hitchcock Medical Center, Lebanon, NH, USA

## Abstract

This letter to the editor responds to Morelli et al, continuing the discussion on the implementation of *DPYD* genotyping.

We agree with Dr. Morelli and colleagues^1^ that logistical challenges are a significant obstacle to wider implementation of *DPYD* genotyping prior to fluoropyrimidine chemotherapy. We applaud these authors for this interesting demonstration that normal (non-malignant) tissue collected during biopsy or resection of cancer can be used as a DNA source for *DPYD* genotyping. This tissue-based approach could serve as a complement to blood-based sample collection in selected circumstances. Another approach that would reduce barriers to acquisition of timely *DPYD* genotype information is widespread implementation of pharmacogenomics in primary care, upstream of a cancer diagnosis.^2^

In closing, we note that the *DPYD* c.2194G>A variant, 1 of 5 variants evaluated by Dr. Morelli and colleagues, is not recognized by the Clinical Pharmacogenetics Implementation Consortium (CPIC) as having an association with reduced DPD enzyme function. CPIC assigns this gene variant as having an activity value of 1 (normal function), which may partly explain the lack of toxicity with this variant in this cohort.
